# Memory Flexibility training (MemFlex) to reduce depressive symptomatology in individuals with major depressive disorder: study protocol for a randomised controlled trial

**DOI:** 10.1186/s13063-015-1029-y

**Published:** 2015-11-03

**Authors:** Caitlin Hitchcock, Emily Hammond, Catrin Rees, Inderpal Panesar, Peter Watson, Aliza Werner-Seidler, Tim Dalgleish

**Affiliations:** Medical Research Council Cognition and Brain Sciences Unit, Cambridge, UK; Cambridgeshire and Peterborough National Health Service Foundation Trust (CPFT), Cambridge, UK; University of Exeter, Exeter, Devon UK; The Black Dog Institute, Prince of Wales Hospital, Randwick, NSW Australia

**Keywords:** Depression, Cognitive flexibility, Autobiographical memory

## Abstract

**Background:**

Major depressive disorder (MDD) is associated with chronic biases in the allocation of attention and recollection of personal memories. Impaired flexibility in attention and autobiographical memory retrieval is seen to both maintain current symptoms and predict future depression. Development of innovative interventions to reduce maladaptive cognitive patterns and improve cognitive flexibility in the domain of memory may therefore advance current treatment approaches for depression. Memory specificity training and cognitive bias modification techniques have both shown some promise in improving cognitive flexibility. Here we outline plans for a trial of an innovative memory flexibility training programme, MemFlex, which advances current training techniques with the aim of improving flexibility of autobiographical memory retrieval. This trial seeks to estimate the efficacy of MemFlex, provide data on feasibility, and begin to explore mechanisms of change.

**Methods/design:**

We plan a single-blind, randomised, controlled, patient-level trial in which 50 individuals with MDD will complete either psychoeducation (n = 25) or MemFlex (n = 25). After completing pre-treatment measures and an orientation session, participants complete eight workbook-based sessions at home. Participants will then be assessed at post-treatment and at 3 month follow-up. The co-primary outcomes are depressive symptoms and diagnostic status at 3 month follow-up. The secondary outcomes are memory flexibility at post-treatment and number of depression free days at 3 month follow-up. Other process outcomes and mediators of any treatment effects will also be explored.

**Discussion:**

This trial will establish the efficacy of MemFlex in improving memory flexibility, and reducing depressive symptoms. Any effects on process measures related to relapse may also indicate whether MemFlex may be helpful in reducing vulnerability to future depressive episodes. The low-intensity and workbook-based format of the programme may improve access to psychological therapies, and, if encouraging, the results of this study will provide a platform for later-phase trials.

**Trial registration:**

NCT02371291 (ClinicalTrials.gov), registered 9 February 2015.

## Background

Major depressive disorder (MDD) runs a chronic and unrelenting course. While current treatments for depression are effective, rates of relapse are high, with more than 80 % of sufferers experiencing future episodes [1]. Attention has therefore turned to how best to enhance and advance current therapeutic practice in order to reduce relapse. Along with the stereotypical symptom of low mood, depression is associated with a range of cognitive impairments [2]. In particular, depression is associated with fixed, habitual biases in the allocation of attention and recollection of personal memories. Chronic cognitive bias, or reduced cognitive flexibility, is seen to both maintain current symptoms and predict future depression [3, 4]. Development of innovative interventions to reduce maladaptive cognitive patterns and improve cognitive flexibility may therefore advance current treatment approaches for depression [3, 5, 6].

Cognitive flexibility is a key contributor to daily wellbeing. The absence of flexibility is linked to a number of different psychopathologies [7], and is an integral feature of depression [8]. Rigid and automatic cognitive bias is most prominent in attending to and remembering information [9]. When attending to information, depressed individuals demonstrate negative inferences about ambiguous material [6], preferential bias towards negative stimuli [3, 6], and a chronic tendency towards negative attributions of the self and world [10]. When remembering information, depressed individuals demonstrate a lack of flexibility in moving between general and specific personal memories [11] and, subsequently, impaired retrieval of specific memories [4]. Attention and memory biases also appear to combine to produce a bias toward the recall of negative information [12]. As rigid cognitive patterns continue to be observed once depression has remitted [13], a lack of cognitive flexibility is considered a vulnerability factor for future depressive symptoms [6].

Empirical attention has therefore turned to interventions that target cognitive flexibility. Attentional bias has been primarily targeted though cognitive bias modification (CBM) [14]. CBM trains individuals to repeatedly engage with neutral or positive stimuli to reduce the automaticity with which negative information is attended to, thereby reducing negative bias. Originally designed to reduce the tendency towards selective processing of threatening information in anxiety, the technique has begun to be applied to depression-related biases. Meta-analysis demonstrates a moderate effect of CBM in reducing both attention and processing biases in depressed samples [14]. Prior research has also suggested a positive impact of CBM on depressive symptoms (e.g. [15]). Though further research is needed, CBM may hold some promise for targeting attention bias in depressed samples.

Changing the nature of retrieved autobiographical (i.e. personal) memories has been the primary target for memory-based interventions. Autobiographical memories may be recalled as specific memories, and refer to one particular event (e.g. having dinner on my birthday this year), or general memories, which summarise categories of events (e.g. my birthday parties). Depressed individuals consistently demonstrate reduced recall of specific memories in cued retrieval tasks, which in turn predicts depression symptoms up to a year later (for review see [16]). Reduced specificity also reduces everyday skills such as problem solving [17] and the ability to readily imagine future events (which is critical for planning) [18]. Memory specificity training therefore aims to improve access to specific memories by educating the participant about the different types and roles of autobiographical memories, and giving them repeated practice in recalling specific memories.

Current evidence for the likely efficacy of training enhanced specificity is promising. The initial training programme devised by Raes and colleagues [19] improved depressive symptoms in a sample of inpatients in an uncontrolled trial. In the first randomised controlled study, Neshat-Doost et al. [20] also demonstrated that bereaved adolescents who completed 5 weeks of memory specificity training experienced fewer symptoms of depression at 2 month follow-up compared to a control condition. Further, the impact of training on depressive symptoms at follow-up was mediated by improvement in memory specificity. Training has also proved successful in those with post-traumatic stress disorder (e.g. [21]), suggesting potential transdiagnostic effects. There are currently further evaluations of memory specificity training underway (e.g. [22]); however, these initial findings indicate that targeting reduced specificity shows promise in alleviating depressive symptoms.

The bias toward negative memories, and corresponding reduced access to positive memories, has also received some attention in interventions. In depressed samples, negative memories are recalled more often than positive memories [12] which contributes to the pervasively negative view of the self and world [9]. When positive memories are recalled, they are less vivid [23, 24], and have a reduced impact on affect [25, 26]. Recall of positive memories is a key technique in regulating negative affect [27] and, as such, reduced access and quality of positive memories is likely to impact mood regulation. CBM techniques for reducing negative attentional bias have begun to be adapted for memory retrieval in depressed samples, and have been seen to reduce negative generalisation and depressive symptoms [28, 29]. Novel approaches toward bias reduction have also emerged. The Method-of-Loci technique has been successfully used as a mnemonic device to facilitate the recall of elaborated positive memories in depressed samples [30]. Reduction of bias toward negative memories and enrichment of positive memories through the adaptation of existing memory therapeutics is therefore likely to aid cognitive flexibility and, in turn, psychological wellbeing.

In sum, depressed individuals demonstrate cognitive inflexibility in their attention to emotional information, along with retrieval of autobiographical memories. Biases in these cognitive processes can be durable cognitive markers of depression, and are related to symptom severity. Previous interventions have yielded positive results, and suggest that training cognitive flexibility may reduce current symptoms. However, current training techniques could be enhanced to improve treatment effects. For example, it is not only impaired recall of specific memories but also the ability to alternate between specific and general memories that relates to depression symptoms [11]. Improving access to specific memories through memory specificity training may influence flexible movement between memory types; however, further direct training of this skill may more effectively improve cognitive flexibility. In addition, flexibility training could be advanced to simultaneously target both the negative and general memory bias, rather than training these skills through separate programmes. This would reduce treatment duration, more holistically train cognitive flexibility, and potentially achieve greater improvement in depressive symptoms. Finally, cognitive models of depression indicate that improved flexibility may reduce the risk of relapse by improving daily functioning skills such as problem solving and mood regulation techniques. However, mechanisms underlying flexibility training have yet to be comprehensively explored. This proposed trial aims to advance current training practices by addressing each of these issues.

We plan to evaluate a novel cognitive intervention, MemFlex. The MemFlex programme combines CBM techniques and memory specificity training to simultaneously target multiple cognitive biases and thereby improve flexibility in three key areas: 1) reducing bias toward negative information in memory and attention; 2) improving the quality of positive memories by encouraging memory elaboration; and 3) encouraging flexible movement between general and specific autobiographical memories. We compare MemFlex to a psychoeducation control condition in a sample of depressed British adults to establish the efficacy and feasibility of MemFlex. We also provide a preliminary examination of whether MemFlex may impact everyday processes such as problem solving, rumination, and cognitive avoidance, and if changes in these processes may underlie treatment effects. In doing so, we address three key questions. First, does MemFlex improve memory flexibility and reduce depression symptoms? Second, is any improvement superior to that achieved by a psychoeducation intervention? Finally, what are the likely mechanisms mediating any treatment effects? We hypothesise a significant effect of treatment condition on our co-primary outcome variables of depressive symptoms and depression status at 3 month follow- up. Specifically, we predict that participants in the MemFlex condition will exhibit fewer depressive symptoms, and be less likely to meet criteria for a major depressive episode than those in the psychoeducation condition. We also hypothesise that participants in the MemFlex condition will demonstrate increased flexibility in memory retrieval at post-treatment and a greater number of depression-free days at 3 month follow-up, relative to the psycheducation control, as secondary outcomes.

## Methods/design

### Study design

The design is a single-blind, patient-level, randomised controlled trial (RCT) comparing MemFlex to psychoeducation. Each condition will comprise a 4-week programme, consisting of an initial face-to-face session, followed by 4 weeks to complete a self-guided workbook. Participants will be assessed pre-treatment, post-treatment, and at a 3 month follow-up. Upon completion of the 3 month follow-up, those in the psychoeducation condition will be given access to the MemFlex materials. The co-primary outcome measures will be depressive symptoms and diagnostic status at 3 month follow-up.

### Participants and recruitment

We will recruit 25 participants per condition (n = 50) with a primary diagnosis of MDD. Inclusion criteria are age over 18 years and currently experiencing a major depressive episode, as assessed using the Structured Clinical Interview for the Diagnostic and Statistical Manual (DSM)-IV (SCID) [31]. Participants will be excluded if their SCID assessment indicates they are experiencing another mood disorder, psychosis, or current alcohol or substance dependence/abuse. Those with a diagnosed personality  disorder or brain injury (assessed by participant report) will also be excluded.

Participants will be recruited in three ways. First, we will recruit from the community via posters and advertisements. Potential participants who make contact after seeing an advertisement will be sent an information sheet. If consent to contact is given, we will then telephone the individual to complete an initial telephone screening. Second, we will recruit from our department volunteer database, which consists of approximately 300 volunteers who have a history of depression and have expressed a wish to participate in research into the disorder. Those on the database will be emailed the study information sheet, and directed to contact the research team if they would like to participate. Again, those interested in participating will then complete an initial telephone screening. Finally, we will also recruit those from clinical facilities who have given consent to be contacted regarding participation in depression research. Recruitment of these participants will follow the same procedure as our database. Following the initial telephone screening, all eligible participants will be invited to complete a SCID and the pre-treatment assessment.

### Participant allocation

**Fig. 1:**
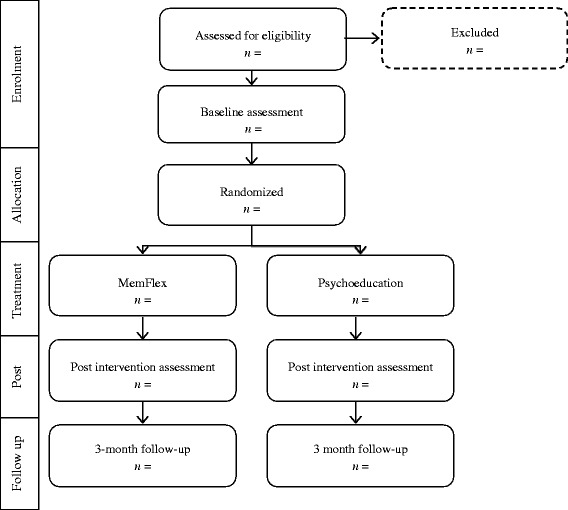
CONSORT diagram

Random allocation to condition using computer-generated, quasi-random numbers will be completed by the trial statistician (PW), who is blind to study objectives. Once a participant has completed the pre-treatment assessment, the researcher completing the initial session will be informed of condition allocation. This process is demonstrated in the CONSORT diagram in Fig. 1.

### Interventions

#### MemFlex

The MemFlex programme draws on CBM and memory specificity training techniques [19, 22]. MemFlex is primarily self-guided and covers three key memory skills to reduce autobiographical memory biases associated with depression. These skills are summarized as 'balancing', 'elaboration', and 'flexibility'. The training material is presented over one face-to-face introductory session and eight self-guided sessions. The initial face-to-face session is completed with a researcher at the research centre. In this session the researcher provides an introduction to different memory systems (e.g. working memory versus autobiographical memory), and outlines the importance of autobiographical memory in everyday life. The impact of depression on autobiographical memory is covered, along with the different types of autobiographical memories (e.g. specific, general). The researcher introduces cued–recall tasks which are used throughout the workbook, and guides the participant in completion of these tasks. When understanding of the basic principles is satisfactory, the researcher assists the participant to set a schedule for completion of the workbook over the following 4 weeks. Participants will receive weekly emails during this period encouraging them to complete the workbook. They will also receive a telephone call from a team member at the beginning of week 3 to check progress, and clarify any difficulties with the workbook material.

Session 1 of the workbook reviews the material covered in the face-to-face session, and introduces the 'balancing' skill. Balancing refers to improving access to both specific and general memories of neutral and positive valence. Following a description of the skill, the participant is given practice retrieving positive and neutral memories. As depression is consistently associated with prolific retrieval of negative and general autobiographical memories, we focus on improving access to other autobiographical memories. Session 2 introduces 'elaboration'; that is, enriching the detail of recalled memories and improving access to the emotion attached to the memory. Again, once the skill is introduced, the participant is given practice elaborating positive memories. Session 3 consolidates previous skills, before 'flexibility' is introduced in Session 4. The flexibility skill helps participant to move between specific and general memories. Sessions 5 to 8 give participants practice in using the different skills, and aim to develop automaticity of the retrieval processes. In addition to completing the sessions in the workbook, individuals are required to practice generative memory retrieval multiple times a day.

We have previously evaluated the feasibility of MemFlex with a group of 38 individuals with a history of MDD but currently in remission. Based on feedback from those individuals, the workbook was refined, and expanded from six to eight sessions. The revised workbook was then subjected to a final feasibility evaluation with three individuals with a current diagnosis of MDD. No further changes were made.

### Psychoeducation

Similar to the intervention condition, participants in the psychoeducation condition will also complete an initial face-to-face session. This session will cover the symptoms and causes of depression, and the workbook will be introduced. As in the MemFlex condition, the workbook will consist of eight self-guided sessions that the individual will be required to complete over 4 weeks. Participants will receive weekly emails during this period encouraging them to complete the workbook. They will also receive a telephone call from a team member at the beginning of week 3 to check progress, and clarify any difficulties with the workbook material.

The workbook content will cover the presentation of depression and basic information on factors associated with depression, such as worry, procrastination, and sleep difficulties. Each session will consist of information on the psychological theory concerning the topic, and an explanation of the impact of the identified factor on depressive symptoms. This will be illustrated with examples. Each session will also include a series of questions about the material to ensure participant engagement.

The workbook content was developed by clinical psychologists (CH and TD). Two additional clinical psychologists independent of the study were consulted regarding the suitability of the workbook material, and their recommended refinements were made. An internal pilot of the workbook was completed with the first three participants allocated to the psychoeducation condition. No further amendments were made.

### Treatment integrity

Initial face-to-face sessions will be manualised and will be completed by clinical staff. All staff will be trained in the administration of the session by a clinical psychologist (CH), who will also provide ongoing supervision. A fidelity checklist will be completed by the therapist at the end of each individual session to assess adherence to the manual. Sessions will also be audio-recorded, and 15 % of these will be independently rated for adherence to the manual. A 2-week extension will be granted if the workbook is not completed within the set 4-week period. That is, up to 6 weeks will be allowed for completion of the workbook. To determine adherence, the researcher will review the workbook at the post-treatment assessment, and record the number of sessions the participant has completed. This will inform our understanding of the acceptability and feasibility of the programme.

### Measures

#### Primary outcomes

The co-primary outcomes are depressive symptoms at 3 month follow-up, as reported on the Beck Depression Inventory II (BDI-II), and diagnostic status at 3 monthfollow-up, which will be measured using the Longitudinal Interval Follow-up Evaluation of the SCID [31].

### Secondary outcomes

Of secondary interest is the number of depression-free days since post-treatment, which will be measured at 3 month follow-up using the Longitudinal Interval Follow-up Evaluation of the SCID [31]. We are also interested in the flexibility of autobiographical retrieval at post-treatment, measured using the Autobiographical Memory Task (AMT) with Alternating Instructions [11]. As MemFlex aims to improve the flexibility with which an individual can move between specific and general memories, we used an alternating instruction AMT (AMT-AI), rather than the traditional AMT [32], which indexes the retrieval of specific memories alone. The AMT-AI requires participants to retrieve specific memories to a block of six cue words, general memories for a block of six cues, and finally a block of twelve cues in which the individual must alternate between retrieval of specific and general memories. Order of the specific and general blocks is randomised between participants. Cues are of positive, negative, and neutral valence, and are also randomised to block. The number and proportion of correct responses is calculated for each block (i.e. specific, general, alternating). The total number of correct responses and the number of correct responses in the alternating block will be used as the main outcome variables.

### Process measures

We will also consider mechanisms through which MemFlex may impact depressive symptoms. Potential mechanisms have been drawn from previous evidence of processes mediating the effect of autobiographical memory on depression (reviewed in [33]). These processes include: 1) problem solving, measured using the short version of the Means-Ends Problem Solving task [34]; 2) rumination, which will be assessed using the Rumination Response Scale [35]; 3) cognitive avoidance, indexed by the Cognitive Avoidance Questionnaire [36]; and 4) verbal fluency as a measure of executive control, as indexed by the Verbal Fluency Task [37]. Each of these measures possesses adequate psychometric properties and has been previously used in evaluation of autobiographical memory interventions. Parallel forms of these measures will be administered at pre- and post-treatment, allowing us to determine whether change in these factors mediates the effect of MemFlex on depressive symptoms at follow-up.

### Methodological aspects

#### Power analysis and sample size

Although a standard power calculation based on detecting treatment effects would be the conventional approach to determining sample sizes for clinical trials, the main aim of the current Phase II trial is to provide a point-estimate of efficacy, and evaluate acceptability and feasibility of MemFlex, in preparation for later phase evaluations of the intervention in line with UK Medical Research Council (MRC) guidance. Our previous experience with Phase II exploratory trial platforms indicates that 50 participants (25 in each arm) will provide sufficient numbers to estimate outcome, and evaluate feasibility and acceptability, and to generate preliminary process data for this treatment. This will provide a plausible range of point estimates of effect on our set of outcome measures sufficient to guide us in sample size calculations for later phase trial work.

### Data collection

All participants will complete assessments at pre-treatment, post-treatment, and at 3 month follow-up. All measures will be administered face-to-face at pre- and post-treatment. The follow-up assessment will involve symptom measures only and will be administered either in person or over the telephone. Assessments will be completed by clinical research staff under the supervision of a clinical psychologist (CH). Inter-rater reliability will be completed for the AMT-AI to ensure high data quality. Data will be securely stored in de-identified form onsite.

### Blinding

The study will employ a single-blind design. This will be achieved by condition allocation not being revealed until the pre-treatment assessment has been completed. Post-treatment and follow-up assessments will be completed by a blinded assessor.

### Statistical analysis plan

All analyses will be completed by the trial statistician, in accordance with CONSORT guidelines. The effect of treatment condition (MemFlex, psychoeducation) on our co-primary outcome of the number of participants who meet criteria for a major depressive episode at follow-up will be determined using chi square analysis. The effect of treatment condition on our co-primary outcome of the number of depressive symptoms at follow-up and on our secondary outcomes will be analysed using independent samples *t*-tests. Baseline differences on relevant measures will be included as covariates, if appropriate. We will also examine mediation effects to determine any mechanisms of change. Assessment of mediation will be completed according to recommendations of Hayes [38]. Multiple imputation will be used to account for missing data. Intent-to-treat analysis will also be used for those lost to attrition. No interim analyses are to be completed.

### Monitoring and data management

This trial will be completed at the MRC Cognition and Brain Sciences Unit in Cambridge. Data checks will be completed by the trial coordinator (CH) to ensure data quality. As this study is an exploratory trial, an independent data monitoring committee was not deemed necessary. All management will be completed by the trial lead (TD), trial coordinator (CH), and trial statistician (PW).

### Safety aspects

No adverse events are anticipated as a result of the study. However, as all participants will be clinically depressed, there is risk of suicidality and self-harm. This will be monitored using clinical judgement and structured risk assessment. Both the trial lead (TD) and coordinator (CH) are clinical psychologists experienced in the treatment and management of depression, and will adhere to MRC safety protocols if adverse events do occur.

### Ethics and dissemination

All participants will provide informed consent prior to participating in the study. Ethics approval has been granted by the UK National Research Ethics Committee (East of England, 11/H0305/1). MRC, National Health Service and professional ethical guidelines will be adhered to throughout the study. No restrictions have been placed on the publication of results.

## Discussion

Due to high relapse rates for depression, clinical research has begun to target cognitive vulnerabilities which may predispose the individual to future episodes. The flexibility of attention and memory represent well-established vulnerabilities, and previous interventions in this area have been promising (e.g. [20, 29]). The MemFlex programme aims to expand existing interventions by simultaneously targeting multiple inflexibilities, and by beginning to explore the mechanisms through which the flexibility of memory retrieval impacts depressive symptoms. The proposed RCT also advances the field by offering a self-guided, non-computer-based version of flexibility training. This format may engage individuals who may not commit to face-to-face therapy, and the low intensity programme may help to improve depressive symptoms to a point where more intensive therapy, such as cognitive behavioural therapy (CBT), may be completed. Relative to group or one-to-one work, self-guided work may also be more easily offered as an adjunct to CBT, or in situations when access to trained mental health care workers is limited. Coupled with the reduced cost of administration, this increases accessibility of treatment, and may see that the programme is both more affordable and more accessible than existing training options.

Evaluation of novel and innovative treatments is vital in optimising the accessibility of evidence-based intervention, and meeting the aims of the Improving Access to Psychological Treatment initiative in the United Kingdom. If the clinical promise of MemFlex can be established, this trial will be a platform for a larger scale RCT. A Phase III trial will allow us to definitively determine the size of treatment effects, and more comprehensively examine the mechanisms underlying these effects. Novel and innovative intervention options present an exciting avenue for research with clinical samples, and such work may ultimately lead to improved accessibility of psychological treatment.

## Trial status

Recruitment began in January 2015.

## Abbreviations

AMT: Autobiographical Memory Task
AMT-AI: Autobiographical Memory Task with alternating instructions
BDI-II: Beck Depression Inventory II
CBM: Cognitive bias modification
CBT: Cognitive behavioral therapy
DSM: Diagnostic and Statistical Manual
MDD: Major depressive disorder
MRC: Medical Research Council
RCT: Randomised controlled trial
SCID: Structured Clinical Interview for the Diagnostic and Statistical Manual-IV
